# Supplementary material for: Heart girth best predicts live weights of market-age pigs in Tanzania
Source: PLoS One. 2023 Dec 6;18(12):e0295433. doi: 10.1371/journal.pone.0295433 (PMC10699594; doi:10.1371/journal.pone.0295433)
Supplement: Attachment — Submitted filename: RESPONSE TO REVIEWERS.docx [file pone.0295433.s003.docx]

**RESPONSE TO REVIEWERS’ COMMENTS**

| **REVIEWER 1** | | |
| --- | --- | --- |
| **SN** | **COMMENT** | **RESPONSE** |
| 1 | My main issue is that this is a crowded space of publication, amazingly. A paper not even cited by the author (<https://link.springer.com/article/10.1007/s11250-023-03561-z>) has effectively already done this study, coming up with a slightly different but entirely analagous result. It is unclear, other than having taken a slightly different approach, how this manuscript builds on that previously done work. Just being specific to Tanzania (and one small region within it) cannot be enough in this context - if the Marshall paper meets the need well, where is the novelty and the added value? | The author appreciates the views of the reviewers. However, the following should be noted;   1. The very first draft of the paper was written during June to August 2022, way before the Marshall’s paper was published. At the time this paper was submitted to PLOS ONE, the author was not aware of the Marshall’s paper, hence could not have cited it. 2. Although this papers bears some similarities with Marshall’s, different approaches have been used. In addition, for this kind of studies, model developed in one area may not be suitable to be used in another area, due to inherent differences between pigs and management practices. Hence a model developed using Ugandan pigs may not be suitable for use in Tanzanian pigs. Hence, findings of this study are still deemed as important to inform tailor-made practice. A point to add is that, the area that the study was carried out is located within the major pig production area in Tanzania with about a half of the pig population; hence the results could more effectively represent the larger pig population |
| 2 |  |  |
| **REVIEWER 2** | | |
| **SN** | **COMMENT** | **RESPONSE** |
| 1 | Line 38: delete -s in ‘involves’ | Comment is received. However the ‘involves’ refers to the ‘pig marketing’, hence it is deemed as being right |
| 2 | Line 64 -68: it can be clarified that there were two objectives that the study sought to address (then show how each was addressed, and the results) | Clarification has been made, as suggested |
| 3 | Line 70: Good to mention that participants willingly consented to participate in the study (were other data collected, besides the measurements?) | A sentence has been added, to mention that farmers ’ willingly participated |
| 4 | Line 93- 105: to make it easier to follow, perhaps have subheadings under 2.3 corresponding to the two objectives – i.e., development of the model, validation of existing weight estimation tape. What informed the sample size of 400? How were the 40 selected, it is mentioned randomly, but not clear how, in the field – was a list of pigs/ households made available before? I suggest we also have a summary description of these 40 | Description of how the 400 and 40 pigs were obtained (for model development and validation of the existing heart girth tape, respectively) has been improved to make it more clear |
| 5 | Line 107: Can include the version used | A mention of STATA version used has been added |
| 6 | Table 1: may be instead of both use ‘all pigs’ | Suggested correction has been made |
| 7 | Table 2: shouldn’t ‘all pigs’ be in the row below (n=400?)? please indicate, in brackets, the numbers for the other rows (pigs below market age, and market age pigs) | Suggested correction has been made |
| 8 | Table 5: please clarify that this is only for the 40 pigs subjected to both weighing, and the tape—the model estimation results, is it for the 40 (not for all pigs)? | Description on how the 40 pigs were obtained and used has been added in the text |
| 9 | References: review all, check number 18, some of the author names are missing | A check has been made and relevant corrections made |
