# Supplementary material for: Heart girth best predicts live weights of market-age pigs in Tanzania
Source: PLoS One. 2023 Dec 6;18(12):e0295433. doi: 10.1371/journal.pone.0295433 (PMC10699594; doi:10.1371/journal.pone.0295433)
Supplement: Attachment — Submitted filename: reviewer comments June28.pdf [file pone.0295433.s002.pdf]

PLOS ONE

Heart girth best predicts live weights of market-age pigs in Tanzania

Dear Editor, PLOS ONE

Thank you for giving me an opportunity to review this manuscript. It is well written, and just had a few comments which I list below.

Kind regards,

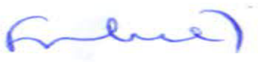

Florence Mutua

Line 38: delete -s in ‘involves’

Line 64 -68: it can be clarified that there were two objectives that the study sought to address (then show how each was addressed, and the results)

Line 70: Good to mention that participants willingly consented to participate in the study (were other data collected, besides the measurements?)

Line 93- 105: to make it easier to follow, perhaps have subheadings under 2.3 corresponding to the two objectives – i.e., development of the model, validation of existing weight estimation tape. What informed the sample size of 400? How were the 40 selected, it is mentioned randomly, but not clear how, in the field – was a list of pigs/ households made available before? I suggest we also have a summary description of these 40

Line 107: Can include the version used

Table 1: may be instead of both use ‘all pigs’

Table 2: shouldn’t ‘all pigs’ be in the row below (n=400?)? please indicate, in brackets, the numbers for the other rows (pigs below market age, and market age pigs)

Table 5: please clarify that this is only for the 40 pigs subjected to both weighing, and the tape—the model estimation results, is it for the 40 (not for all pigs)?

References: review all, check number 18, some of the author names are missing
